# *Phaser.MRage*: automated molecular replacement

**DOI:** 10.1107/S0907444913022750

**Published:** 2013-10-18

**Authors:** Gábor Bunkóczi, Nathaniel Echols, Airlie J. McCoy, Robert D. Oeffner, Paul D. Adams, Randy J. Read

**Affiliations:** a Department of Haematology, University of Cambridge, CIMR, Wellcome Trust/MRC Building, Addenbrooke’s Hospital, Hills Road, Cambridge CB2 0XY, England; b Lawrence Berkeley National Laboratory, One Cyclotron Road, Mailstop 64R0121, Berkeley, CA 94720, USA

**Keywords:** molecular replacement, pipeline, automation, *phaser.MRage*

## Abstract

*Phaser.MRage* is a molecular-replacement automation framework that implements a full model-generation workflow and provides several layers of model exploration to the user. It is designed to handle a large number of models and can distribute calculations efficiently onto parallel hardware. In addition, *phaser.MRage* can identify correct solutions and use this information to accelerate the search. Firstly, it can quickly score all alternative models of a component once a correct solution has been found. Secondly, it can perform extensive analysis of identified solutions to find protein assemblies and can employ assembled models for subsequent searches. Thirdly, it is able to use *a priori* assembly information (derived from, for example, homologues) to speculatively place and score molecules, thereby customizing the search procedure to a certain class of protein molecule (for example, antibodies) and incorporating additional biological information into molecular replacement.

## Abstract 2

The functionality of the molecular-replacement pipeline *phaser.MRage* is introduced and illustrated with examples.

## 1. Introduction

Molecular replacement is by far the most frequently used method for solving the phase problem, accounting for nearly 80% of the structures deposited in the Protein Data Bank (Berman *et al.*, 2003 ▶) in 2012. One factor that has contributed to its overwhelming popularity is the rapid growth of available structures that provide an increasingly complete structural coverage of protein families. In addition, advances in automatic model building, especially the integration of structure modelling in, for example, *phenix.mr_rosetta* (DiMaio *et al.*, 2011 ▶) and *AMPLE* (Bibby *et al.*, 2012 ▶), have increased the radius of convergence for refinement and led to successful structure determination from borderline molecular-replacement solutions. Simultaneously, improvements in the accuracy of homology detection (Jaroszewski *et al.*, 2005 ▶; Söding *et al.*, 2005 ▶) and developments in model improvement (Schwarzenbacher *et al.*, 2004 ▶; Bunkóczi & Read, 2011 ▶) have enabled the reliable identification of more distant homologues and improved their applicability as suitable molecular-replacement models.

In a general case, when solving a structure by molecular replacement many alternative models need to be tried, but increased numbers of potential molecular-replacement models can make manual execution of searches tedious and in some cases unfeasible. Automation of molecular replacement and integration with homology detection and model improvement is a promising solution to this problem (Long *et al.*, 2008 ▶; Keegan & Winn, 2008 ▶; Keegan *et al.*, 2011 ▶; Stokes-Rees & Sliz, 2010 ▶).

Although access to computing resources has improved significantly over the last two decades, the size of molecular-replacement problems can still be overwhelming, and therefore it is important to automate the process in an efficient manner. *Phaser.MRage*, a recently developed molecular-replacement automation program, uses an artificial intelligence approach to organize and execute searches. In this paper, the architecture and design goals are introduced and the functionality is illustrated with examples.

## 2. Implementation

The controlling logic is based on the blackboard pattern (Buschmann *et al.*, 1996 ▶), which is commonly used in artificial intelligence applications. In short, the ‘blackboard’ is a common knowledge base, which initially contains the problem specification and is iteratively updated by a group of semi-autonomous specialists (‘knowledge sources’) to arrive at a solution (http://en.wikipedia.org/wiki/Blackboard_system). There are no hardwired processing logics; knowledge sources are activated opportunistically when there is a contribution to make. Therefore, the blackboard pattern makes it particularly simple to incorporate new manipulation steps or change the solution strategy, even at runtime. On the other hand, a notable weakness of the blackboard pattern is that it cannot handle concurrency. Therefore, it is combined with the master–slave pattern (Buschmann *et al.*, 1996 ▶), which delegates CPU-intensive calculations to child processes and thus offloads the artificial intelligence layer. An additional advantage of this pattern is that it hides the actual mode of execution and allows it to be replaced without any changes to other components. *Phaser.MRage* uses a recently developed open-source library module in the *Computational Crystallography Toolbox* (*cctbx*; Grosse-Kunstleve *et al.*, 2002 ▶) that allows programs to use multiple processors (CPUs or CPU cores) either in the local machine or in clusters accessible through batch submission queues (Bunkóczi & Echols, 2012 ▶, 2013 ▶). Popular batch queue systems (including Sun Grid Engine and Portable Batch System) are supported and are fully customiz­able from the command line (contributions or requests for novel systems are also welcome). Deployment onto clusters requires no additional setup, apart from making the installation accessible from each node and specifying custom options for the submission command line (for example, submission to a particular queue). All communication with the spawned subprocesses is handled internally either through a network-based or a file-system-based channel, and from the user perspective setting up a multiprocessor job only requires selecting the number of CPUs and the execution medium.

### 2.1. Calculations

*Phaser.MRage* uses *Phaser* (McCoy *et al.*, 2007 ▶) to perform molecular-replacement calculations. Functionality is exported *via* Boost.Python bindings (Abrahams & Grosse-Kunstleve, 2003 ▶) and is used *via* Python (http://www.python.org) function calls. Algorithmic improvements in the *Phaser* molecular-replacement code are therefore immediately available to *phaser.MRage* and there is no need for code duplication.

While the implementation is possible to replace, *phaser.MRage* does depend on some properties inherent to the maximum-likelihood molecular-replacement calculation. One particularly important property is that scores may be directly compared, *i.e.* if two models are evaluated against the same data the better one gives a higher score. This is exploited at several points in the decision making.

### 2.2. Parallel processing

Instead of using the MR_AUTO mode of *Phaser*, *phaser.MRage* runs the steps of molecular replacement separately, which has several advantages. Firstly, this allows resource allocation to grow linearly with the complexity of the search, *i.e.* after each branch point the number of independent jobs created equals the degree of branching. Therefore, given unlimited resources and an ideal computing environment, the simplest molecular-replacement search containing one model and one clear peak and a complex one containing several models and a high degree of branching would take the same time. Secondly, this allows resource reallocation to searches that are progressing more slowly than others.

Certain steps in molecular replacement (*e.g.* the packing function) can be faster than the overhead of starting a child process. To overcome this issue, *phaser.MRage* supports the pooling of fast calculations and packaging them into a single job. Although this reduces the degree of parallelism, it increases the efficiency of the search as a whole.

### 2.3. Dependencies and availability

*Phaser.MRage* is built around *Phaser* (McCoy *et al.*, 2007 ▶) and crystallographic algorithms provided by *cctbx* (Grosse-Kunstleve *et al.*, 2002 ▶). It is currently distributed with the *PHENIX* package (Adams *et al.*, 2010 ▶). The program can be run on a wide range of commodity hardware starting with a simple laptop, although for moderately complex searches a multi-core workstation is recommended but is not essential. Support for queuing systems allows it to scale to hundreds of processors on managed clusters.

*Phaser.MRage* can be run either by preparing an input file or through the *PHENIX* GUI (Echols *et al.*, 2012 ▶). A tutorial introduction (explaining input preparation through the GUI, but also showing the resulting command script) is available at http://www.phaser.cimr.cam.ac.uk/index.php/Molecular_replacement_with_MRage.

## 3. Functionality

Automated molecular replacement is a complex process involving a range of methods from bioinformatics to crystallo­graphy. Although a perfect automation framework would offer a complete collection of tools available for each workflow step, it is clear that such a system would not be feasible either from the maintenance (requiring frequent updates to follow changes in all the dependencies) or from the system-administration (managing external dependencies, for example homology-search software) point of view. However, restricting users at these steps to a few choices made by the authors is also not an optimal strategy, because those methods could be superseded and limit the utility of the whole system. For this reason, *phaser.MRage* employs a mix of these two extremes. Firstly, molecular-replacement and model-editing calculations are limited to those provided by the *PHENIX* package (Adams *et al.*, 2010 ▶). Secondly, when an external tool is used a default option is provided, with the choice of default sometimes guided by convenience rather than power. However, in these steps users are given the opportunity to use other external tools, input the results and bypass the built-in tool. Results can be input to the program in popular formats which are relatively well established (requiring less maintenance) and also potentially ubiquitous (output from many external tools can be converted to this format).

A good example is homology search. *Phaser.MRage* offers the possibility to perform a homology search using either a locally installed *BLAST* executable (Altschul *et al.*, 1990 ▶) or through the NCBI web service (http://blast.ncbi.nlm.nih.gov/Blast.cgi). However, *BLAST* is not optimal for identifying or aligning weak homologues (lower than 25% identical to the target). Alternatives are, for example, *PSI-BLAST* (Altschul *et al.*, 1997 ▶), *FFAS* (Jaroszewski *et al.*, 2005 ▶) and *HHpred* (Söding *et al.*, 2005 ▶), which all require up-to-date databases and local installations are therefore not easy to manage. However, all of these are available through web servers and (with the exception of *FFAS*) their output can be input to *phaser.MRage*. This naturally requires that users perform the initial step manually, but it also enables them to employ the most appropriate tool for the problem at hand.

### 3.1. Input

**Figure 1:**
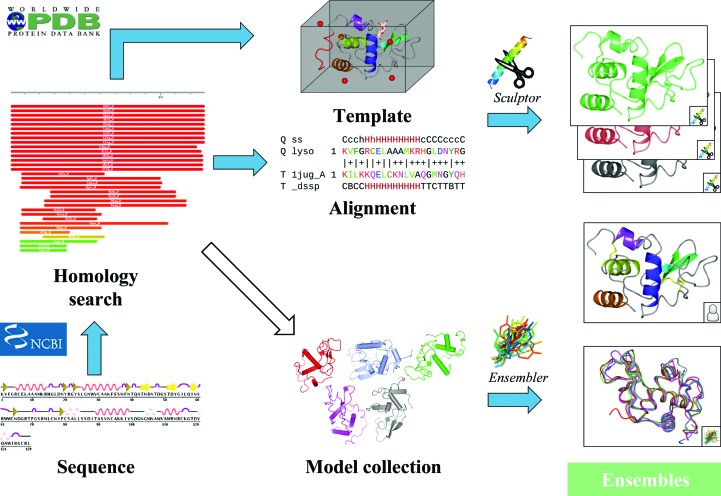
*Phaser.MRage* workflow showing the model-generation hierarchy. The Ensembles stage (indicated with white text on a light green background) is used directly in molecular-replacement calculations. Blue arrows indicate existing processing steps. The empty arrow highlights a possible automation step that could select models for a multi-model ensemble from a set of hits detected by a homology search. Users can select models using any combination of the displayed stages, and the highlighted steps will be performed to convert those into the Ensembles stage. The molecular graphics in this figure were rendered with *CCP*4*mg* (McNicholas *et al.*, 2011 ▶).

*Phaser.MRage* requires an X-ray data set and a description of potential models to run; for novice users, this will most often be the protein sequence. It can handle arbitrary numbers of copies of an arbitrary number of components. The description of models is organized into a hierarchy of processing stages for each search component. Stages higher on the hierarchy are more generic and can lead to several lower, more specialized, stages. Therefore, a single input file at the top-level processing stage can lead to hundreds of potential search models (Fig. 1 ▶).

(i) *Ensemble*. This is the bottom stage of the processing hierarchy and involves no further processing. This model will be used without modification. It is expected that it covers the full sequence of the search component, but there is no other requirement.
(ii) *Model collection*. This is a set of models that can be converted into a multi-model ensemble. The program *Ensembler*, which is also distributed as a standalone application with the *PHENIX* (Adams *et al.*, 2010 ▶) and *CCP*4 (Winn *et al.*, 2011 ▶) packages, is used to perform superposition and optional trimming.
(iii) *Model template*. This is the structure of a homologue that can be used as a molecular-replacement model after improvement. If no sequence alignment with the target is provided, an alignment will be made using *MUSCLE* (Edgar, 2004 ▶). *Sculptor* (Bunkóczi & Read, 2011 ▶) will be used to convert these to a number of ensembles, depending on the selected sculpting protocols (by default, all protocols will be tried). *Phaser.MRage* will also use the alignment to determine which parts of the target sequence are modelled by the homologue.
(iv) *Homology-search result*. The result of a homology search against the sequence of the component. Output is accepted from several popular homology-search programs including *BLAST* (Altschul *et al.*, 1990 ▶), *PSI-BLAST* (Altschul *et al.*, 1997 ▶) and *HHpred* (Söding *et al.*, 2005 ▶). It is processed into a number of model templates, depending on the number of hits, extracting the alignments contained in the homology search.
(v) *Sequence*. This is used to define the scattering power of the component. In addition, it can be used to perform a homology search, which has to be requested explicitly or it will not take place.

If there are two or more components, the estimated stoichiometry in the putative complex needs to be provided. All model descriptions specified for a search component will be used as alternative models, but more specialized inputs are given priority in the search. It is also valid to specify a component sequence with no models or homology search requested; indeed, this is necessary so that it can be taken into account for composition calculations.

The asymmetric unit content can either be specified or left for the program to establish, in which case the program will select the most likely composition based on the Matthews coefficient, taking into account all components with the specified stoichiometry. If this is an overestimate, the program can still identify the correct composition, albeit with some added computing overhead. In the case of underestimated composition, the search will be finished after reaching the requested number of complexes, therefore leading to a partial solution.

### 3.2. Extension-cycle processing steps

**Figure 2:**
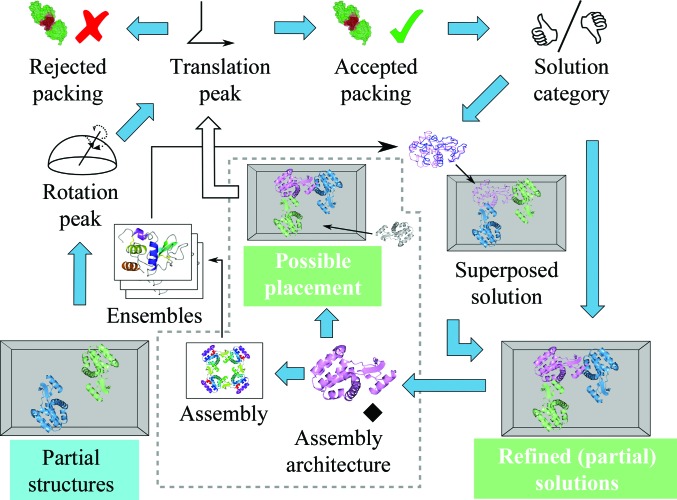
Simplified molecular-replacement workflow of an extension cycle, including solution analyses. The process starts with partial solutions taken from the previous cycle (marked with a light blue box) and ends with refined solutions that will be propagated to the next cycle (marked with white text on a light green background), if any. Common molecular-replacement steps are performed with each model that is applicable to a given partial structure (decided by the composition). If a clear solution is identified, quick scoring by superposition can be performed. The dead-end ‘Rejected packing’ is shown to highlight a potential automation step, namely automatic model pruning, if rejected solutions are found with good statistics. The grey dashed box highlights solution-analysis steps. Assemblies identified after refinement (or specified by the user) are used to fill in missing molecules (also shown as white text on a light green background), which enter the workflow as a translation peak (in the following cycle; indicated by the empty arrow). Assemblies can also be used to augment the model list (if requested). With the exception of solution categorization and steps in solution analysis, all processing steps indicated by arrows can run in parallel. The molecular graphics in this figure were rendered with *CCP*4*mg* (McNicholas *et al.*, 2011 ▶).

The program runs a series of extension cycles, in which it tries to extend active partial solutions (or an initial empty solution in the first round) with all compatible models. After each extension cycle, it checks whether there are any new results. If no possible extension is found for any of the extendable partial models, the search is finished. This can arise if no components are missing from the current partial solutions (*i.e.* a full solution is found) or if all possible extended models are rejected (*e.g.* on packing grounds). The workflow is shown schematically in Fig. 2 ▶.

#### 3.2.1. Composition analysis

At the beginning of each extension cycle, for each partial structure located in the previous step *phaser.MRage* establishes the composition missing with respect to the defined contents of the asymmetric unit. It then collects all defined or potential models (such as templates and homology-search hits) and checks whether or not they are contained in the missing composition. This is performed using a sequence-based algorithm. For templates and homology-search hits the associated alignment is taken into account to determine which segments are covered. Models that are acceptable on composition grounds are then marked and will be used for molecular-replacement searches.

#### 3.2.2. Homology search and data fetch

If the sequence of the component is specified and a homology search is requested, the program will perform a *BLAST* search (Altschul *et al.*, 1990 ▶) using either a local installation or at the NCBI site (http://blast.ncbi.nlm.nih.gov/Blast.cgi). Additional services with suitable programmatic interfaces may be integrated in the near future.

Template PDB files for homology-search hits are fetched from the wwPDB (Berman *et al.*, 2003 ▶). These are then paired up with the corresponding alignment from the homology search to create a model template.

#### 3.2.3. Model editing and ensembling

Model templates need to be improved before they are used in molecular replacement (Schwarzenbacher *et al.*, 2004 ▶). *Phaser.MRage* uses *Sculptor* (Bunkóczi & Read, 2011 ▶) to process the templates. Protocols for pruning atoms and adjusting *B* factors are selected by the user. While in all other cases strict ordering is possible based on model quality, there is only very limited *a priori* knowledge about the relative performance of different protocols and therefore the program tries them in a random order that may change between executions of the program.

*Phaser.MRage* uses *Ensembler* to superpose predefined sets of models (*i.e.* model-collection input) and optionally to trim the resulting ensemble. Because procedures for optimally selecting ensemble components from homology-search hits have yet to be established, models obtained from homology searches are used individually in molecular-replacement searches. Pre-made ensembles can also be input directly and are used without further processing.

#### 3.2.4. Molecular replacement

The workflow is organized into the standard *Phaser* rotation-function, translation-function and packing-function steps, in order of increasing priority. Owing to the priority of evaluation, this leads to a depth-first traversal of the molecular-replacement search tree, while reversing the priorities would result in a breadth-first traversal (http://en.wikipedia.org/wiki/Tree_traversal). Although breadth-first has certain unique advantages, such as the availability of scores for all children of a given node, which enables accurate pruning of low-scoring peaks, in the current setting depth-first traversal is potentially more efficient because it enables the identification of clear solutions early on, which can save significant amounts of computer time. The steps are executed independently and can be run in parallel.

In the presence of a solution prediction (*e.g.* from solution analysis performed in the previous extension cycle; see §[Sec sec3.5]3.5), the program creates the solution and calculates a score. If the created solution is judged to be significant by the solution-identification procedure, the program can bypass the search phase and go directly to adding the next component.

#### 3.2.5. Peak categorization

Each translation peak that passes the packing stage is evaluated to determine whether or not it is a clear solution. This is currently based on high values of the translation-function *Z*-score (McCoy *et al.*, 2005 ▶). Clear solutions are automatically selected for refinement and also participate in post-search analyses, even if they fail to pass the threshold score before the refinement step (Fig. 2 ▶). However, propagation to the next stage is strictly determined by the log-likelihood score from *Phaser* (McCoy *et al.*, 2007 ▶). This feature is designed to retain solutions found for minor components that are overshadowed by good results obtained for larger components and to combine them with partial solutions without performing a search.

#### 3.2.6. Refinement and peak propagation

After all searches have been completed, the program finds the best-scoring peak based on the log-likelihood score from *Phaser* (McCoy *et al.*, 2007 ▶). Peaks failing the packing test are excluded from the selection. A threshold score is determined by calculating the score improvement for the best-scoring peak in the current extension cycle and subtracting a percentage of its absolute value from the best score, and all peaks above the threshold are selected. These are then subjected to refinement. An additional thresholding step is performed to reduce the number of partial structures if a peak refines significantly better than the others, and selected peaks are propagated to the next extension cycle as partial structures.

### 3.3. Solution strategies

*Phaser.MRage* currently offers two strategies or modes. These both include basic model-preparation (Fig. 1 ▶) and molecular-replacement (upper part of Fig. 2 ▶) steps. Although the actions performed are not identical, these both give the same results if no clear solutions are found in the categorization step and only differ after the first clear solution is obtained.

(i) In ‘full’ mode, the traversal of the search tree continues with the current workflow until it is complete.
(ii) In ‘quick’ mode, the traversal of the search tree is terminated once a clear solution is found (although it is important to note that even in this mode multiple clear solutions may be found, for example, if the search employs multiple CPUs). The strategy is modified by removing the rotation and translation functions from the workflow (the packing function is not removed, because it is believed to be fast enough that there is more value in processing already obtained translation peaks compared with the time saving gained by discarding them). In addition, the procedure performing quick rescoring with alternative models is activated.

The ‘quick’ mode can lead to significant time savings. *Phaser.MRage* prioritizes all calculations in decreasing probability of success and therefore the clearest solutions are expected from the calculations near the top of the list, which are processed first. In addition, it can prevent a combinatorial explosion of clear solutions at the expense of calculating an approximation to the best solution (see §[Sec sec4.2.2]4.2.2, aminopeptidase example). In addition, it also allows more exhaustive searches to be run by default, which would explore weaker peaks if necessary, but not affect the runtime if a clear solution is found.

It would theoretically be possible to allow users to define custom strategies at runtime, but this would increase the user-­interface complexity significantly. In addition, a separate analysis step would need to be performed to check whether the defined strategy is functional. On the other hand, a wider selection of preset strategies can be added to the existing ones in order to cater for custom scenarios, and this is the current direction of future development.

### 3.4. Evaluation of alternatives

After a clear solution has been found, it is possible to generate an equivalent solution for all alternative models of the same component by superposition (provided there is sequence overlap), which can subsequently be scored. However, the structure of homologues can differ considerably (Chothia & Lesk, 1986 ▶), especially where functional conformational changes are present. In addition, an optimal superposition based on structure is not identical to that based on electron density, which would be more adequate for the problem at hand. For this reason, refinement of rotational and translational parameters for the model to be evaluated needs to be performed in order to obtain a meaningful score. The only exception seems to be models sharing the same template (generated using different *Sculptor* protocols), which can be scored without refinement.

Superposition is performed using secondary-structure matching (Krissinel & Henrick, 2004 ▶), as it is fast and accurate. If superposition fails, the model is not evaluated.

This technique is very efficient in finding the best model from a series of alternatives. In addition to saving time, it also overcomes a search artefact, namely that the search is performed on a finite grid. The solutions generated are marked as clear solutions irrespective of the score.

### 3.5. Solution analyses

After an extension cycle is completed, the program analyses clear solutions (identified in the peak-categorization step) to scavenge useful information (such as solution-ancestry and molecular-assembly relations) that may speed up the search for missing components. It is assumed that the solutions analysed are correct but potentially incomplete. The analysis step can either predict a more complete solution or find a more complete model (Fig. 2 ▶).

#### 3.5.1. Amalgamation

This procedure exploits the fact that two clear solutions originating from the same partial solution only differ in the molecules added in the last extension cycle, but otherwise both of them are correct. Therefore, it is possible to combine them into a more complete solution. This is performed by creating an association between the solutions. At the beginning of the next extension cycle, *phaser.MRage* checks for these associations and performs a quick packing and scoring job, followed by solution identification. If the association results in a clear solution the search can be skipped and the resulting more complete solution is propagated. If the starting partial solution is empty, the origin is not defined and the program tries all possible origin shifts for nonpolar space groups. For polar space groups, the rotation is extracted and only the rotation search is skipped.

#### 3.5.2. Assembly identification and completion

The program analyses all molecules related by noncrystallographic symmetry (NCS), transparently transforming related alternative models, to determine whether they are related by an operation that can be a member of a point group. All such operations and point groups are collected and identical groups and subgroups are merged.

The program then takes each molecule that can participate in an assembly and checks whether all possible members of the assembly are present. If not, the missing position is associated with the current solution. At the beginning of the next extension cycle, the program scores these predictions. If one of the predictions turns out to give a clear peak, the search can be skipped and the results propagated to refinement.

In addition, full model assemblies can be used in the search. This can potentially improve the signal by increasing the size of the model searched. Assembly models are used as rigid bodies when performing rotation-function/translation-function calculations, but are disassembled at the refinement stage.

As solutions become more complete, the NCS operators become more precise and require updating. This is also performed at this stage. Point groups of known assemblies are compared with freshly established point groups and equivalent ones are updated.

In addition, it is possible to input known assembly information. This is not restricted to homomeric assemblies and does not have to obey point-group symmetry. If a member of a known assembly has been located with a clear signal, the program generates all missing members for evaluation in the next step. However, the assembly can be imprecise and is often outside the convergence radius of refinement (this is in contrast to the automatically determined assemblies, since these are observed in the current structure). Therefore, the program performs a local search around the predicted position and picks the highest scoring orientation and position.

### 3.6. Space-group identification

*Phaser.MRage* allows three levels of space-group uncertainty: (i) the exact space group is known; (ii) enantiomorph ambiguity; and (iii) only the point group is known. Molecular replacement is performed in all possible space groups compatible with the user choice and the results are compared after each extension cycle. The best solution among all space groups is selected and a threshold is calculated. Space groups whose best solution is above the threshold are propagated with all current partial solutions to the next cycle. This algorithm aims at delaying space-group selection until a clear choice can be made. In the case that no space group proves to be clearly superior to others, the program produces results for all active space groups.

### 3.7. Reducing excessive branching

The exponential growth of potential alternatives after each branching point would make all but the simplest molecular-replacement calculation impractical. For this reason, *phaser.MRage* utilizes the branching-with-pruning strategy also employed by *Phaser* (McCoy *et al.*, 2007 ▶) to keep the number of active branches down to a manageable level. This is used after each extension cycle to select peaks for refinement and also for propagation of peaks after refinement.

Although the above strategy works well when there is only a small number of good solutions, it can break down when there are several good alternative models, as can be the case when all *Sculptor* protocols are used for model improvement and all protocols result in accurate models with comparable quality. In this case, growth of the search tree can be reduced by equating models generated from the same template, classifying spatially equivalent solutions as symmetry equivalents and keeping only the one with the highest score. Template matching may be extended to cover all alternative models of the same component, as long as a meaningful superposition can be made.

### 3.8. Output solution selection

After the extension cycles have completed, the program outputs the best potential solutions. These are selected from the set of all peaks located in all extension cycles based on the associated log-likelihood score. Thresholding is performed as described previously. In addition, solutions that can be regarded as subsets of a better solution (taking crystallo­graphic symmetry into account) are identified and discarded. This way, when the composition is overestimated, the model with the correct number of components will possibly still have the highest score and therefore will not be merged into any other solutions. However, solutions containing additional incorrect molecules (provided that these are not discarded on packing grounds) will also be listed with the best solution but associated with a lower score. Incomplete solutions on the way to the full solution are merged into the full solution and removed from the list, thereby providing a clearer picture. Therefore, the correct and complete solution is still found and output, although the search itself is not interrupted to save computer time. A procedure that addresses the opposite scenario (*i.e.* when the composition is underestimated) has not yet been implemented.

### 3.9. Result handling

Instead of writing out individual PDB and MTZ files for each solution, *phaser.MRage* uses a custom internal format (based on the Python standard library pickle module) to store all solutions found and provides a utility program (*phaser.MRage.solutions*) to access these. This is a deliberate design choice for three reasons. Firstly, it is not possible to know in advance how many solutions will be found. If the number of solutions output is limited and a solution needs to be examined that was not output, one needs to either extract this information from the log file manually or rerun the search requesting a higher number of solutions to output. A potentially better alternative is to store all solutions found in a storage-efficient format and access these with a utility program.

Secondly, this allows extra processing to be performed on the results without rerunning the search, even algorithms that are implemented after the search has finished. Candidates are CPU-intensive calculations that are not an integral part of the molecular-replacement workflow but are sufficiently commonly performed, for example autobuilding.

Thirdly, the internal format does not incur information loss. When using multi-model ensembles, limitations of the current PDB format do not allow the outputting of a solution with full information content in a general case. Therefore, the program offers several options to deal with solutions containing multi-model ensembles and allows users to inspect multiple representation of the same solution. The possibilities are as follows.

(i) Instead of containing several alternative models, it can reformat each multi-model ensemble as a chain with multiple alternative conformations.
(ii) It can score each member of the ensemble against the X-­ray data and select the one with the highest score.
(iii) It can create a ‘chimera’ by pairwise combination of constituent models using *phenix.combine_models* (Adams *et al.*, 2010 ▶) based on the electron-density map.

In addition to PDB format, *phaser.MRage.solutions* can output solutions in several other formats, including XML for easy integration and *Phaser* solution files for search continu­ation.

## 4. Discussion

### 4.1. Comparison with existing software

*BALBES* (Long *et al.*, 2008 ▶; Keegan *et al.*, 2011 ▶) and *MrBUMP* (Keegan & Winn, 2008 ▶; Keegan *et al.*, 2011 ▶) are existing molecular-replacement pipelines that are in common use. Both of them are available in the *CCP*4 suite (Winn *et al.*, 2011 ▶) and approach automation from a different angle.

#### 4.1.1. Architecture

Both *BALBES* and *MrBUMP* delegate molecular replacement to the underlying *Phaser* (McCoy *et al.*, 2007 ▶) and/or *MOLREP* (Vagin & Teplyakov, 2010 ▶) binaries. While this can have advantages, it makes resource reallocation and coordination very difficult. For example, if the search with one model takes much longer than all other searches, idle resources cannot be reallocated to the underlying process. In addition, if a solution has been found with one model it is very difficult to communicate this information to ongoing searches, since there is normally no support for it in the underlying program.

#### 4.1.2. Domains

Both *BALBES* and *MrBUMP* can decompose homology-search hits into underlying domains. However, *phaser.MRage* does not currently use domain-boundary information explicitly. On the other hand, it does benefit from domain-boundary information implicitly available through homology searches. Frequently, a homology-search hit only covers a single domain from a multi-domain protein. This is taken into account by assigning the sequence covered to the homologue *via* a sequence alignment. The assembly algorithm takes the modelled sequence into account and can combine homologues modelling distinct (with some tolerance) parts of the sequence.

#### 4.1.3. Assemblies

*BALBES* records known assembly information in its internal database, while *MrBUMP* can query the *PQS* service at the EBI (http://www.ebi.ac.uk). The detected assemblies are then used as regular models in the search. However, the treatment of multimeric assemblies in *phaser.MRage* is quite different. Assemblies are either found by automatic solution analysis or input by the user. Although it is possible to perform searches with these model assemblies, better results can be achieved by using these with the automatic solution-completion algorithm. The underlying problem is that although tertiary structure is fairly well conserved among models sharing only 30% sequence identity, quaternary structure tends to be more variable; therefore, assemblies found for homologue structures may not be good models for the target structure. *Phaser.MRage* overcomes this problem using two strategies. Firstly, it uses local information from the assembly: it takes located molecules as anchors and places the missing molecules accordingly. In the case where the full assembly is not well preserved, but locally it is within the convergence radius of refinement, extension will succeed. Secondly, assemblies located during solution analyses are representative of the current structure and are therefore potentially transferable to further copies within the asymmetric unit.

#### 4.1.4. External scoring

Quality scores in both *BALBES* and *MrBUMP* are derived from refinement. *MrBUMP* can also start autobuilding for potential solutions found in the procedure, as this is a very powerful way to identify the correct one. *Phaser.MRage* itself does not perform additional scoring calculations apart from calculating the log-likelihood gain for each solution, but subsequent scoring actions could be implemented into the utility program *phaser.MRage.solutions*.

#### 4.1.5. Solution identification

All three systems have their own criteria for identifying correct solutions. *BALBES* and *MrBUMP* use more global criteria, such as refinement *R* factors, while *phaser.MRage* uses the translation-function *Z*-­score, which is related to the significance of a peak to the variance of the corresponding translation search. While this score does not order solutions by quality, it can also be used successfully to identify incomplete partial solutions and therefore allows the program to analyse these at an earlier stage.

### 4.2. Examples

#### 4.2.1. Trypsin

Orthorhombic trypsin (PDB entry 1hj9, one molecule in the asymmetric unit; Leiros *et al.*, 2001 ▶) was solved using a single template (PDB entry 2b9l, 32% identical; Piao *et al.*, 2005 ▶) and an alignment from *ClustalW*2 (Larkin *et al.*, 2007 ▶). All available protocols were used from *Sculptor*, which resulted in 13 models.

**Figure 3:**
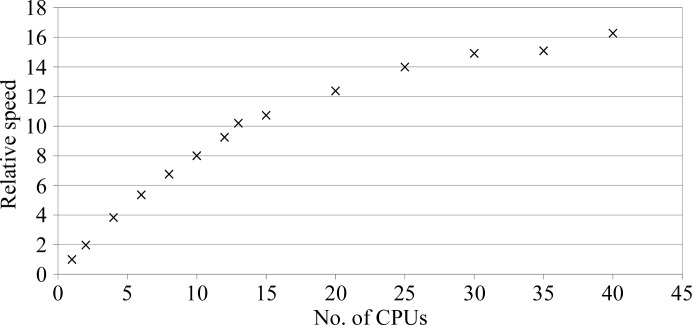
Speedup of the trypsin example (full mode) relative to execution on a single CPU (total time 41 min 30 s). Timings are single measurements performed on a 64-CPU machine. Parallel jobs were run on separate threads. Speedup factors were not corrected for the input-processing (including anisotropic scaling) and job-startup overhead.

Benchmarking was performed on a 64-core multi-CPU machine in ‘full’ mode (to ensure that the exact same searches are processed) to assess resource scaling. The results in Fig. 3 ▶ show that near-linear speedups were found for up to 13 CPU cores (the same as the number of alternative models) and a lower, but still measurable, speedup was found above that. This is a consequence of parallelization at the function level, which aligns resource scaling to the complexity of the search. For very simple problems, scaling could completely stop at a single CPU. However, even moderately difficult problems normally require hundreds of non-dependent calculations which could be run in parallel.

Structure solution was also attempted in ‘quick’ mode. This gave variable timings because models from the same template are processed in random order. About half of the models (protocols 7–12) gave clear solutions with translation-function* Z*-scores in excess of 7.0, while the others were comparatively worse, yielding solutions with translation-function *Z*-scores of about 5 that were sometimes buried in the noise. This reiterates the need to test several *Sculptor* protocols in molecular replacement (Bunkóczi & Read, 2011 ▶). Time savings were very significant when running on a small number of CPUs compared with the full mode (a factor of six for a single CPU) and yielded the same solution list, but diminished quickly with multiple CPUs.

#### 4.2.2. Aminopeptidase

XXA-Pro aminopeptidase (PDB entry 3ovk, four molecules in the asymmetric unit; Midwest Center for Structural Genomics, unpublished work) was solved using a single template (PDB entry 3qoc, 28% identical; Midwest Center for Structural Genomics, unpublished work). All available protocols were used from *Sculptor*.

From the second molecule onwards, all 13 models yielded clear solutions with high *Z*-scores. However, this creates the problem that there are 13^4^ = 28 561 possible quasi-equivalent complete solutions. There are possibilities to contain the combinatorial explosion. In ‘quick’ mode, processing terminates after the first clear solution is found. Therefore, in each extension cycle only the best partial solution (with the highest log-likelihood gain) will be processed (strictly speaking only when running on a single CPU), but all models will be evaluated through superposition. Therefore, this technique leads to the best combination of the best partial structure and all possible models. Similarly, when using template equivalence, all quasi-equivalent solutions are matched as symmetry equivalents at each stage and only the one with the highest score is propagated. This is equivalent to finding the best combination of all partial solutions with all possible models. It is important to note that none of the results are necessarily equivalent to the globally best combination (this can only be found by testing all possibilities), but it is a comparatively good-quality one and reduces the number of calculations to 13 × 4 = 52.

#### 4.2.3. Shiga-like toxin

This crystal form of shiga-like toxin (PDB entry 1bos; Ling *et al.*, 1998 ▶) contains four pentamers in the asymmetric unit. A single 100% sequence-identical monomer model was used from another shiga-like toxin structure (PDB entry 1bov; Stein *et al.*, 1992 ▶). *Phaser.MRage* was able to find all 20 molecules in a relatively short time. This was aided by the presence of pentamers, which were identified early on and were used to fill in the missing chains, thereby skipping many extension phases. When executing the search on multiple CPUs, the search path taken is affected by the precise timing of when a result is received from a worker. In certain search paths, *phaser.MRage *managed to use a full pentameric assembly and fill in five molecules at a time. A summary of such a run is shown in Table 1 ▶.

#### 4.2.4. Glutathione synthase

**Figure 4:**
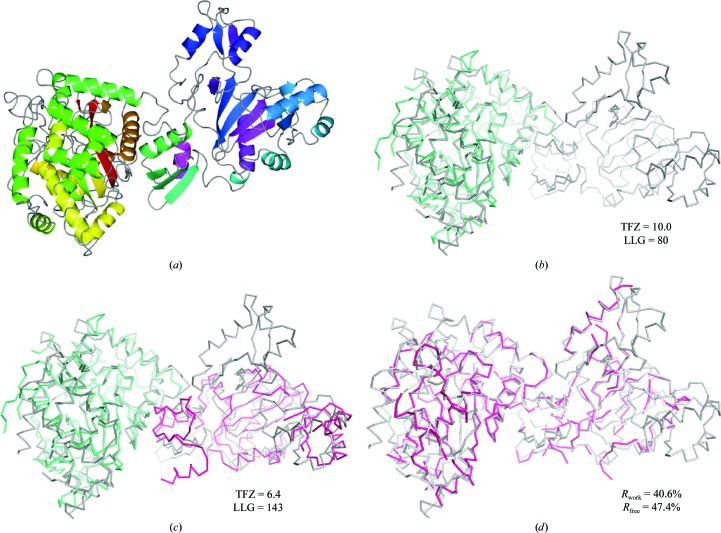
Solution process for the glutathione synthase example: (*a*) target structure, (*b*) N-terminal domain found (PDB entry 3nzt; hit 4; 29% identical), (*c*) C-­terminal domain found (PDB entry 1uc8; hit 7; 21% identical), (*d*) structure after *phenix.autobuild* (Terwilliger *et al.*, 2008 ▶). The grey trace indicates the correct structure. This figure was created using *PyMOL* (v.1.6; Schrödinger LLC).

The structure (PDB entry 3ln6; J. Stout, D. De Vos, B. Vergauwen & S. N. Savvides, unpublished work) is a 750-residue single-chain protein containing two domains. Molecular replacement was started from an *HHpred* (Söding *et al.*, 2005 ▶) search that found 82 hits. All 13 *Sculptor* protocols were used. *Phaser.MRage* relatively quickly located one domain with good statistics using hit 3nzt (Center for Structural Genomics of Infectious Diseases, unpublished work) and then tested all possibilities to find the missing domain. A correct solution was eventually found using PDB entry 1uc8 (Sakai *et al.*, 2003 ▶). The solution is shown in Fig. 4 ▶.

This search was run on a managed cluster and 50 CPUs were allocated. The total runtime was approximately 1 d, which is equivalent to nearly two months of CPU time on a single-CPU machine. This could be reduced by implementing equivalence of (superposable) alternative models, but the processing demand is still considerable. This scale of exhaustive searches can only be performed meaningfully on computing clusters. On the other hand, running the search did not require any human intervention apart from performing an initial *HHpred* search. In addition, the cost in manpower of solving the structure manually is potentially much higher than that of the required computing time. This suggests that given sufficient processing power, a significant fraction of eventually successful molecular-replacement searches could be automated.

## 5. Conclusions

With the increasing availability of relatively high-performance computing resources, automated high-throughput molecular replacement is becoming more prominent in crystallographic structure solution. It is therefore important that automation software is able to handle common molecular-replacement scenarios and to offer a clear advantage to users over manually executing the same programs.

*Phaser.MRage* has been shown to be able to solve a wide range of molecular-replacement problems. When making use of built-in intelligence it can solve simple problems quickly, but it can also handle more complex searches with increasing calculation demands. Efficiency improvements are made possible by the economy of scale and full control over the molecular-replacement process. In addition, integrated tools are used effectively and the possibility of misuse by non-experts is reduced.

Although the program provides an up-to-date workflow with currently state-of-the-art molecular-replacement protocols, it is likely that improved tools and protocols will appear in the near future and may require large-scale changes to the workflow, which will then need to be updated. Therefore, *phaser.MRage* is not tied to any particular workflow, but rather to a set of rules that are believed to be well established and less likely to change. Incorporation of new ideas requires changes to this set of rules and is therefore not disruptive to the program architecture.

## Figures and Tables

**Table 1:** Course of the structure solution for shiga-like toxin (four pentamers, using a monomer as a search model) Significant solutions appear when placing the second copy. As the solution becomes more and more complete, the program identifies a pentameric assembly, adds it to the list of search models and uses it to locate a full pentamer with very clear statistics. Note the low translation-function *Z*-score obtained for the last molecule, which is a consequence of its high *B* factors. It is difficult to locate this molecule using conventional searches and it requires a very thorough exploration. However, when placed in an approximately correct location predicted from available assembly information it is found immediately.

Index	Model	TFZ†	LLG‡	ΔLLG§
1	Monomer	5.3	43.1	43.1
2	Monomer	11.9	154.7	111.6
…				
10	Monomer	22.3	2005.9	293.3
11	5 × monomer	42.6	3889.3	1883.4
16	Monomer	33.5	4545.4	656.1
…				
19	Monomer	38.1	6557.1	673.9
20	Monomer	9.2	7322.9	765.8

† Translation-function *Z*-score.

‡ Log-likelihood gain.

§ Change in LLG from previous step.
